# Reversion of chronic to episodic migraine in working age and botulinum toxin‐resistant patients treated with fremanezumab: A real‐life study

**DOI:** 10.1002/brb3.3631

**Published:** 2024-07-21

**Authors:** Juan Tudela‐Tomas, Rosa‐Maria Ramos‐Guerrero, Maria‐Eugenia Rodriguez‐Mateos

**Affiliations:** ^1^ Servicio de Farmacia Hospitalaria Hospital Universitario Puerta del Mar Cadiz Spain

**Keywords:** calcitonin gene‐related peptide, chronic migraine, fremanezumab (Thermo Fisher Scientific Cat# MA5‐42117, RRID:AB_2911260), migraine reversion, real‐life study

## Abstract

**Objectives:**

The objectives of this real‐life study were to analyze the reversion of chronic migraine (CM) to episodic migraine (EM) with fremanezumab, evaluate its benefit on the symptomatology, and determine the influence of possible clinical features on the reversion.

**Background:**

The clinical manifestations of CM have a high impact on the quality of life of patients, and monoclonal antibodies such as fremanezumab are used as prophylactic treatment.

**Methods:**

Diagnosed CM patients treated for at least 3 months with monthly fremanezumab were interviewed. The data to assess efficacy were before treatment and at the time of the interview: monthly headache days (MHDs), daily headache hours (DHHs), monthly symptomatic medication days (MSMDs), percentage of patients with symptomatic medication overuse (SMO), and pain intensity with the numerical rating scale (NRS) score. Possible predictors of reversion were analyzed: percentage of patients treated for at least 12 months, hypertension, diabetes mellitus, depression, anxiety, symptomatic control with non‐steroidal anti‐inflammatory drugs (NSAIDs), triptans or both, and amitriptyline prophylaxis.

**Results:**

A total of 54 patients were included, of whom 40 (74.1%) were converters to EM. There were significant improvements in converters compared to pre‐treatment in MHDs (28.0 vs. 5.0 days), as well as on the variables DHHs, MSMDs, and SMO. The percentage of erenumab failures was significantly higher in non‐converters than in converters, as was the percentage of patients with anxiety.

**Conclusions:**

High reversion from CM to EM was achieved with fremanezumab and notable symptomatological improvement, establishing previous failure to erenumab and anxiety as possible detrimental factors for reversion.

## Graphical abstract

The objectives of this real‐life study were to analyze the reversion of chronic migraine (CM) to episodic migraine (EM) with fremanezumab. A total of 54 patients were included, of whom 40 (74.1%) were converters from CM to EM. There were significant improvements in converters compared to pre‐treatment in monthly headache days (28.0 vs. 5.0 days), as well as on the variable's daily headache hours, monthly symptomatic medication days, and symptomatic medication overuse. The percentage of erenumab failures was significantly higher in non‐converters than in converters, as was the percentage of patients with anxiety.

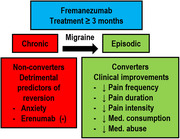

## 1 INTRODUCTION

Migraine is the second leading cause of disability worldwide and the leading cause of years of life lost due to disability in working age women (Steiner et al., 2020).

Clinically, a classification is made depending on the frequency of migraine attacks, which allows it to be categorized as episodic migraine (EM) or chronic migraine (CM), based on the number of days per month with a headache. EM is defined as <15 migraine days per month, while criteria for the diagnosis of CM includes headaches for >15 days per month for at least 3 months and suffering this pain with migraine criteria for at least 8 days (Olesen et al., 2006).

Prophylactic treatments to prevent the occurrence of migraine attacks are widely used in clinical practice, including beta‐antagonists, topiramate, or botulinum toxin (Onabotulinumtoxin A) injections (Diener et al., 2011). The involvement of calcitonin gene‐related peptide (CGRP) in the pathophysiology of migraine led to the development of inhibitory antibodies to this neuropeptide, used as prophylactic treatment, such as fremanezumab, which binds to CGRP, preventing it from binding to its receptor (Edvinsson, 2019). In Spain, the use of fremanezumab is restricted to patients with eight or more migraine days per month, and three or more failures of previous treatments used at sufficient doses for at least 3 months, one of these treatments being botulinum toxin, in the case of CM.

Given the clinical presentation, the use of treatments for symptomatic control is common, including non‐steroidal anti‐inflammatory drugs (NSAIDs) and drugs in the triptan family, among others (Bigal et al., 2009). Failure to control the pain can lead to a high risk of treatment abuse, with a counterproductive effect on the practice (Lipton et al., 2019, 2013). Indeed, CM accompanied by symptomatic medication overuse (SMO) is considered the most common headache (Vos et al., 2015).

As for clinical manifestations accompanying migraine, the relationship between depression and migraine has often been studied, with a possible bidirectional relationship between the two (Breslau et al., 2003), as well as between anxiety and migraine, given the increased emotional stress in migraine patients, and the influence of stress as a trigger for migraine episodes (Wacogne et al., 2003).

Patients with CM present a marked impairment of their daily activities, given the chronicity and high presence of migraine, leading to greater functional and quality of life deterioration (Bigal et al., 2003).

Studies evaluating the reversion of CM to EM by prophylactic treatment with anti‐CGRP antibodies in working age patients are scarce and, more specifically, no real studies with fremanezumab are currently available to evaluate this potential benefit.

The primary objective of this study was to analyze the reversion of CM to EM in working age and botulinum toxin‐resistant patients treated with fremanezumab. The secondary objectives were: (i) determine the influence of possible clinical characteristics of patients on the reversion from CM to EM; (ii) assess the benefits of fremanezumab in the pathology of working age CM patients.

## 2 METHODS

### 2.1 Study design and patients

A real‐life observational study was performed with patients aged 18–65 years diagnosed with CM and under neurological follow‐up, who were treated with fremanezumab (Thermo Fisher Scientific Cat MA5‐42117, RRID:AB_2911260)for a minimum of 3 months. The study was approved by the Ethics Committee for Research with Medicines of the Province of Cadiz (Protocol Number 14022, approved on January 26, 2023), and written informed consent was requested from all participating patients. All patients recruited met the selection criteria of the positioning reports for the Spanish Agency of Medicines and Medical Devices.

Digital health records were searched to identify patients treated with fremanezumab as a 225 mg monthly injection. Patients who were not diagnosed with CM were excluded, as well as those who had not been treated for at least 3 months with this drug. Patients who could not give consent on their own were also excluded from the study, as well as those over 65 years of age, given the intention to target a working‐age population. This treatment period is justified according to the authorization results of fremanezumab, which proved superior effectiveness to placebo in the prophylaxis of episodes in patients with CM after 3 months of treatment (Silberstein et al., 2017).

The interview process was carried out in the pharmaceutical offices of the Puerta del Mar Hospital Cadiz, Spain, starting on February 13, 2023, and ending on April 28, 2023. All data collected were stored in an anonymous database.

### 2.2 Effectiveness measures

In the interviews with each patient, the following variables were collected to assess their possible influence on reversion from CM to EM: sex, age, duration of treatment with fremanezumab, previous failure with erenumab, symptomatic control with triptans, symptomatic control with NSAIDs or combined with both, use of amitriptyline as prophylactic treatment, hypertension, type 1 diabetes mellitus, depression, and anxiety.

As to the length of the treatment with fremanezumab, it was decided to reflect the percentage of patients that had been treated for >12 months, considering that this was the cutoff point for data collection in reversion studies published in the past (Altamura et al., 2022). It was decided to collect if a switch from erenumab to fremanezumab had been made to assess the possible influence on reversion of previous failures to monoclonal antibodies with a different mechanism of action.

The diagnosis of hypertension was chosen because it is a pathology traditionally studied concomitantly with migraine and with possible influences in the process of chronification (Barbanti et al., 2010).

Interest in the diagnosis of depression was based on the fact that it is a common comorbidity in migraine, especially in CM, and that fremanezumab is so far the only drug against CGRP to show improvement in depressive status (Lipton et al., 2021).

Including the diagnosis of type 1 diabetes mellitus was decided based on the inverse association between diabetes mellitus and migraine prevalence (Hagen et al., 2018).

Only patients with a diagnosis of hypertension, type 1 diabetes mellitus, depression, or anxiety, diagnosed by a specialist or primary care physician, and with current prescription of antihypertensive, hypoglycemic, antidepressant, or anxiolytic treatment, respectively, were considered valid.

To assess the reversion from CM to EM, the number of monthly headache days (MHDs) before starting treatment with fremanezumab and at the time of the interview was used as the primary variable. Converters were defined as those patients whose number of MHDs decreased to <15 days after at least 3 months of fremanezumab treatment. Patients who did not reach this target were defined as non‐converters.

The remaining effectiveness variables were assessed before starting fremanezumab treatment and at the time of the interview, selecting number of daily headache hours (DHHs), number of monthly symptomatic medication days (MSMDs), percentage of patients with SMO, and headache intensity with numerical rating scale (NRS).

The NRS was developed in 1978 and is widely used for its sensitivity and the auditability of its results (Karcioglu et al., 2018). It ranges from 0 to 10, representing the lowest to the highest possible pain, respectively. Patients were instructed to mark the mean value they experienced during a month, both before starting treatment and at the time of the study.

The criterion for considering strong medication use as abusive was set at taking medication at least 15 days a month (Olesen et al., 2006).

The data that support the findings of this study are available on request from the corresponding author.

### 2.3 Statistical analysis

To study reversion, median MHDs, DHHs, MSMDs, percentage of patients with SMO, and NRS score were compared between converters and non‐converters. The percentage of patients with at least 12 months of treatment, hypertension, diabetes mellitus, depression, anxiety, symptomatic control with NSAIDs, triptans or both, and prophylactic treatment with amitriptyline were also compared. Medians were plotted with their 95% confidence interval (CI), and comparisons were made using the Mann–Whitney *U* test. Contingency tables were constructed for percentage comparisons of categorical variables and the two‐tailed Fisher's exact test was applied. The level of statistical significance was set at *p* < .05. All statistical analyses were performed with SPSS 27.0 (SPSS Inc.).

## 3 RESULTS

### 3.1 Clinical and demographic data

**FIGURE 1:**
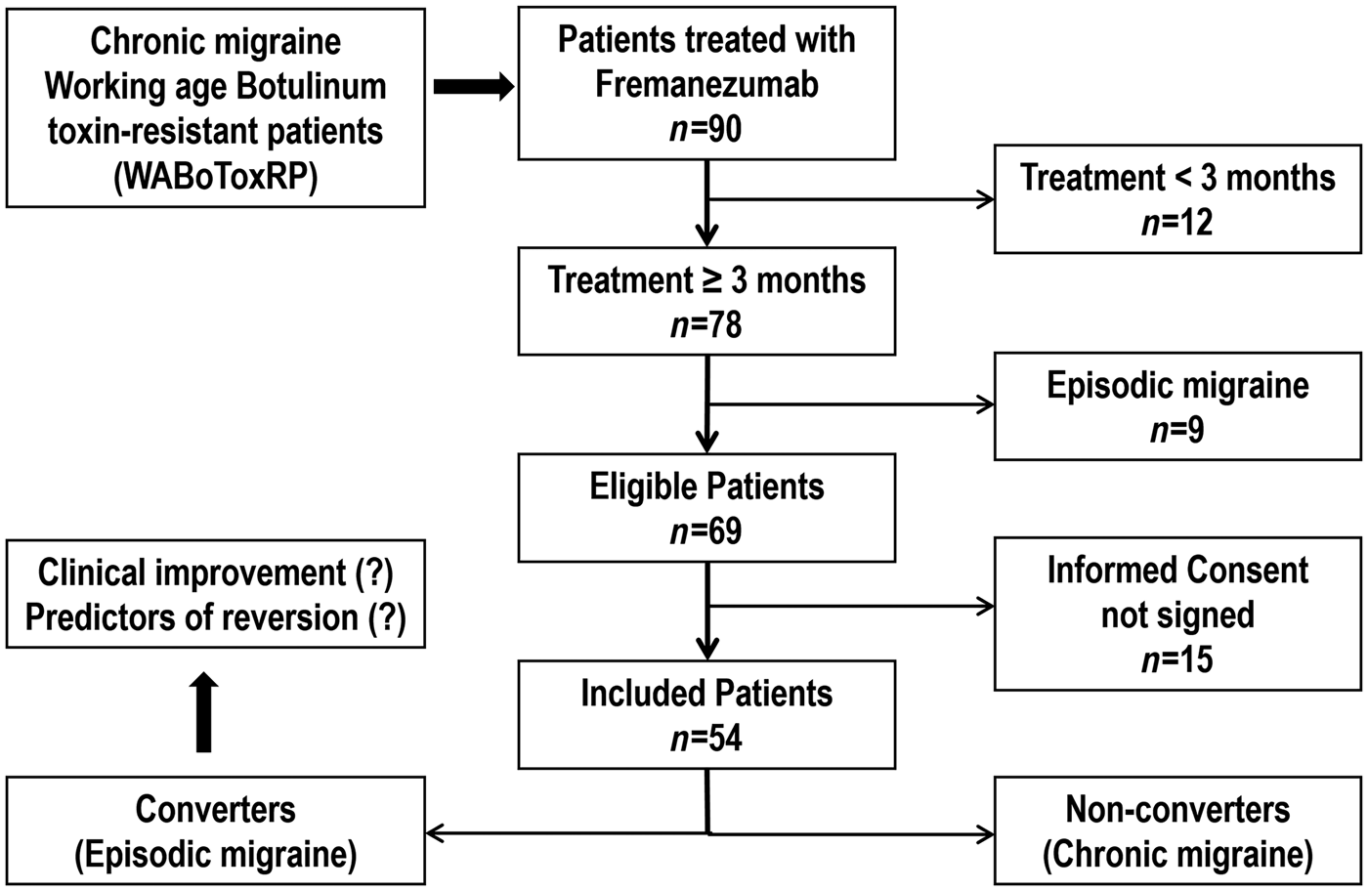
Flow chart of the real‐life study.

A total of 90 potentially eligible patients treated with fremanezumab were identified through digital medical records, of which 12 were rejected because they had not taken the medication for at least 3 months. Nice were rejected because they had a diagnosis of EM. Of the 69 eligible patients, 15 did not sign the informed consent form and thus did not participate in the study. In the end, a total of 54 patients were included in the study (Figure 1).

Demographic and clinical data were collected for all patients in the study (Table 1). The median age of the study population was 51.5 years old (95% CI, 47.4–55.3), with a median follow‐up period of 12 months and a median treatment duration of 12 months (95% CI, 9.4–15.0).

**TABLE 1:** Clinical and demographic characteristics of the study population.

Characteristics (total patients = 54)	Data
Female sex (*n*; %)	50 (92.6%)
Age, years (median, 95% CI)	51.5 (47.4–55‐3)
Treatment duration, months (median, 95% CI)	12.0 (9.4–15.0)
≥12 Months treatment (*n*; %)	31 (59.3%)
Baseline MHDs (median, 95% CI)	28.0 (28.0–29.0)
Baseline DHHs (median, 95% CI)	24.0 (21.0–24.0)
Baseline MSMDs (median, 95% CI)	30.0 (28.3–30.0)
Baseline SMO (*n*; %)	52 (96.3%)
Baseline NRS (median, 95% CI)	8.0 (8.0–9.0)
Previous erenumab failure (*n*; %)	9 (16.7%)
Symptomatic medication (*n*; %)	
•Triptans	44 (81.5%)
•NSAID	38 (70.4%)
•Combined	30 (55.6%)
Amitriptyline prophylaxis (*n*; %)	16 (29.6%)
Hypertension (*n*; %)	11 (20.4%)
T1DM (*n*; %)	3 (5.6%)
Depression (*n*; %)	20 (37.0%)
Anxiety (*n*; %)	20 (37.0%)

Abbreviations: CI, confidence interval; DHHs, daily headache hours; MHDs, monthly headache days; MSMDs, monthly symptomatic medication days; NRS, numerical rating scale; NSAIDS, non‐steroidal anti‐inflammatory drugs; SMO, symptomatic medication overuse; T1DM, type 1 diabetes mellitus.

According to baseline patient characteristics (Table 2), the total number of converting patients was 40 (74.1%) and non‐converting patients was 14 (25.9%). The percentage of patients with previous erenumab treatment failure was lower in converters than in non‐converters patients (10.0% vs. 35.7%, *p* = .041). The percentage of patients with a diagnosis of anxiety was lower in converters than in non‐converters (27.5% vs. 64.3%, *p* = .024). No statistically significant differences were detected in the other baseline characteristics between converters and non‐converters.

**TABLE 2:** Clinical and demographic characteristics of converters versus non‐converters.

Characteristics	Converters (*n* = 40)	Non‐converters (*n* = 14)	*p* value
Female sex (*n*; %)	36 (90.0%)	13 (92.9%)	0.991
Age, years (median, 95% CI)	52 (49.4–56.6)	45 (40.0–58.1)	0.077
Treatment duration, months (median, 95% CI)	13.5 (11.4–16.0)	8.5 (7.9–14.1)	0.080
≥12 Months treatment (*n*; %)	26 (65.0%)	5 (35.7%)	0.068
Baseline MHDs (median, 95% CI)	28.0 (23.8–28.0)	29.0 (29.0–30.0)	0.074
Baseline DHHs (median, 95% CI)	23.5 (22.0–24.0)	24.0 (21.0–24.0)	0.627
Baseline MSMDs (median, 95% CI)	29.0 (25.8–30.0)	30.0 (28.0–30.0)	0.067
Baseline SMO (*n*; %)	39 (97.5%)	13 (92.9%)	0.274
Baseline NRS (median, 95% CI)	8.0 (8.0–8.5)	9.0 (9.0–10.0)	0.065
Previous erenumab failure (*n*; %)	4 (10.0%)	5 (35.7%)	0.041
Symptomatic medication (*n*; %)			
•Triptans	34 (85.0%)	10 (71.4%)	0.424
•NSAID	29 (72.5%)	9 (64.3%)	0.735
•Combined	24 (60.0%)	6 (42.9%)	0.353
Amitriptyline prophylaxis (*n*; %)	9 (22.5%)	7 (50.0%)	0.087
Hypertension (*n*; %)	7 (17.5%)	4 (28.5%)	0.448
T1DM (*n*; %)	2 (5.0%)	1 (7.1%)	0.982
Depression (*n*; %)	12 (30.0%)	8 (57.1%)	0.108
Anxiety (*n*; %)	11 (27.5%)	9 (64.3%)	0.024

Abbreviations: CI, confidence interval; DHHs, daily headache hours; MHDs, monthly headache days; MSMDs, monthly symptomatic medication days; NRS, numerical rating scale; NSAIDS, non‐steroidal anti‐inflammatory drugs; SMO, symptomatic medication overuse; T1DM, type 1 diabetes mellitus.

### 3.2 Treatment effectiveness

**FIGURE 2:**
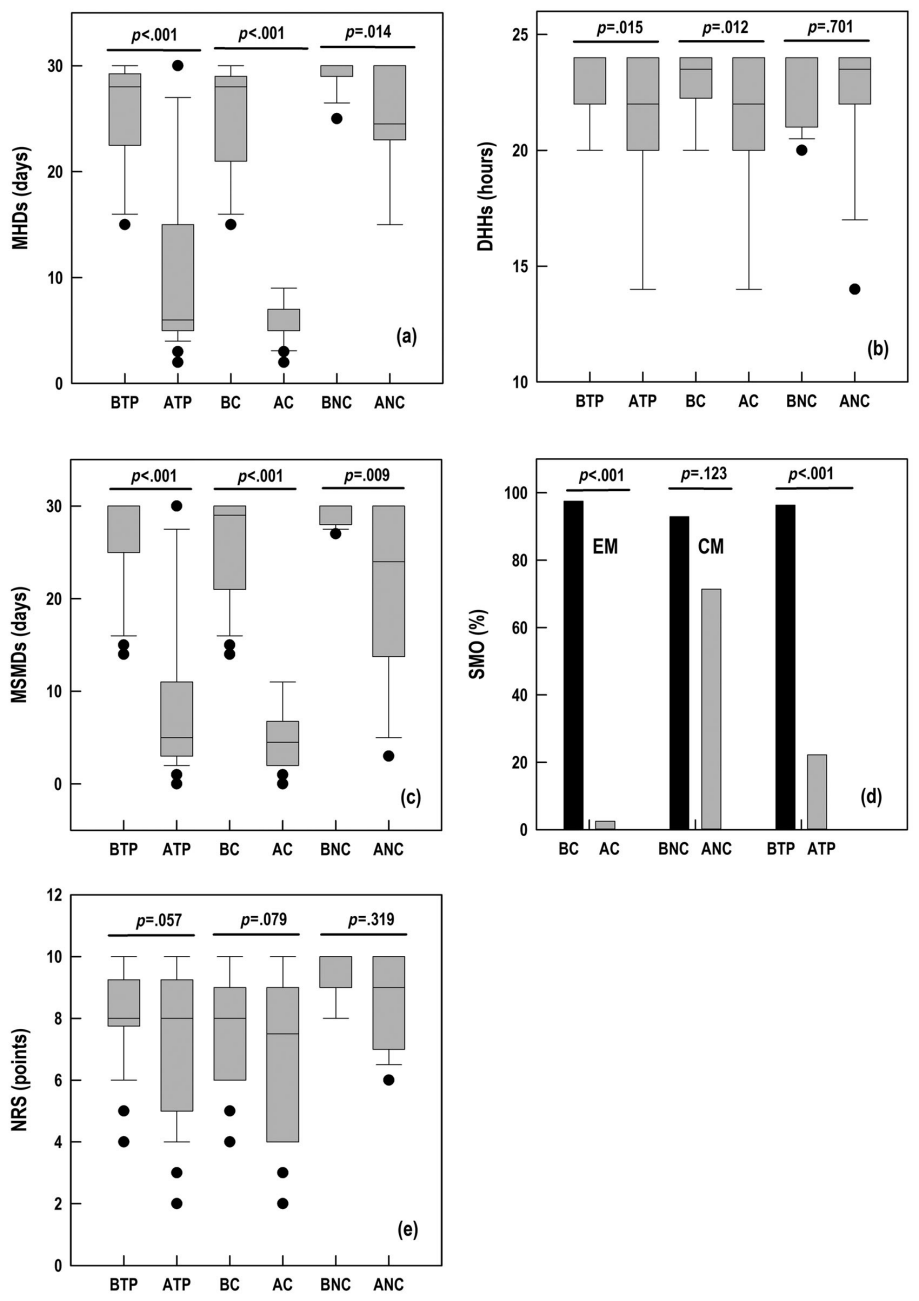
Evolution of disease in patients after fremanezumab treatment in converters and non‐converters. Box plots (a, b, c, and e): (a) MHDs, monthly headache days data, (b) DHHs, daily headache hours data, (c) MSMDs, monthly symptomatic medication days data, (e) NRS, numerical rating scale score data. *X* axis, Box plots: The boundary of the box closest to zero indicates the 25th percentile, a line within the box marks the 50th percentile (median), and the boundary of the box farthest from zero indicates the 75th percentile. Box lines: Whiskers (error bars) above and below the box indicate the 90th and 10th percentiles. Points: Outlier values are depicted as data points. Bar chart (d): Percentage of patients with SMO, symptomatic medication overuse. Bars of median values. AC, actual converters; ANC, actual non‐converters; ATP, actual total population; BC, baseline converters; BNC, baseline non‐converters; BTP, baseline total population.

The median of MHDs (Figure 2a) fell from 28.0 (95% CI, 28.0–29.0) to 6.0 (95% CI, 5.0–8.0; *p* < .001) in the total population, while it decreased from 28.0 (95% CI, 23.8–28.0) to 5.0 (95% CI, 5.0–6.0; *p* < .001) in converters and from 29.0 (95% CI, 29.0–30.0) to 23.0 (95% CI, 23.0–30.0; *p* = .014) in non‐converters.

The median of DHHs (Figure 2b) fell from 24.0 (95% CI, 21.0–24.0) to 22.0 (95% CI, 22.0–23.0; *p* = .015) in the total population, while it decreased from 23.5 (95% CI, 22.0–24.0) to 21.5 (95% CI, 18.0–24.0; *p* = .012) in converters and from 24.0 (95% CI, 21.0–24.0) to 23.5 (95% CI, 22.0–24.0; *p* = .701) in non‐converters.

The median of MSMDs (Figure 2c) fell from 30.0 (95% CI, 28.3–30.0) to 5.0 (95% CI, 4.0–8.0; *p* < .001) in the total population, while it decreased from 29.0 (95% CI, 25.8–30.0) to 4.5 (95% CI, 3.0–5.0; *p* < .001) in converters and from 30.0 (95% CI, 28.0–30.0) to 24.0 (95% CI, 14.7–30.0; *p* = .009) in non‐converters.

The percentage of patients with SMO (Figure 2d) fell from 96.3% to 22.2% (*p* < .001), while it decreased from 97.5% to 2.5% (*p* < .001) in converters and from 92.9% to 71.4% (*p* = .123) in non‐converters.

The median NRS score (Figure 2e) fell from 8.0 (95% CI, 8.0–9.0) to 8.0 (95% CI, 7.0–8.0; *p* = .057) in the total population, while it decreased from 8.0 (95% CI, 8.0–8.5) to 7.5 (95% CI, 5.0–8.0; *p* = .079) in converters and from 9.0 (95% CI, 9.0–10.0) to 9.0 (95% CI, 7.0–10.0; *p* = .319) in non‐converters.

## 4 DISCUSSION

The high percentage of women stands out (90.0% vs. 92.9%, *p* = 0.991) in converters and non‐converters (Tables 1 and 2). The prevalence of migraine in women has been widely described, and the influence of estrogen, whose variations can act as a trigger, is well known, as early as puberty, and at times such as pregnancy and pre‐menopause (Lay & Broner, 2009). Since no significant differences were found between the two groups, the possible influence of sex as a predictor of CM to EM reversion was ruled out.

As for median age (Tables 1 and 2), there were no statistically significant differences between converters and non‐converters. Age has been ruled out as a possible predictive factor in influencing the reversion of CM to EM (Seok et al., 2006).

It was detected (Tables 1 and 2) that the percentage of patients treated for at least 12 months was slightly higher, although not statistically significant, in converters compared to non‐converters (65.0% vs. 35.7%, *p* = .068). Given the effectiveness of clinical results with fremanezumab, reported studies of CM to EM reversion with fremanezumab are limited to 3 months in duration (Ashina et al., 2019; Cohen et al., 2018; Lipton et al., 2020; Singh et al., 2018). The results of this study call for a 12‐month follow‐up period study, as done with galcanezumab (Altamura et al., 2022).

The percentage of patients with previous erenumab failure (Tables 1 and 2) was significantly lower in converters compared to non‐converters (10.0% vs. 35.7%, *p* = .041). The results seem to show a poorer prognosis in CM patients after switching from erenumab to fremanezumab (Overeem et al., 2022).

The diagnosis of anxiety (Tables 1 and 2) was higher in the non‐converted population, and significantly higher than in the converted population (64.3% vs. 27.5%, *p* = .024). Anxiety is a more frequent comorbidity in patients with CM than with EM, and uncontrolled anxiety can lead to a worsening of their baseline pathology (Buse et al., 2013).

The need for amitriptyline prophylaxis (Tables 1 and 2) was lower in a non‐statistically significant way for non‐converters than in converters (22.5% vs. 50.0%, *p* = .087), which in the absence of further studies could be indicative of a possible predisposing factor to non‐reversion in patients with more symptomatology.

The use of fremanezumab had a remarkable impact on the symptomatology of the studied patients. The main effectiveness variable, MHDs (Figure 2a), decreased statistically significantly in converters, with reversion achieved in the majority (50 out of 64) of patients. These results in working age and botulinum toxin‐resistant patients support the effectiveness of fremanezumab in the treatment of CM obtained in clinical trials (Ashina et al., 2019; Cohen et al., 2018; Lipton et al., 2019; Singh et al., 2018).

The decrease in converters of other effectiveness variables, DHHs (Figure 2b), MSMDs (Figure 2c), and NRS (Figure 2e), shows a high benefit in patients' clinical and quality of life, while the large decrease in the percentage of patients with SMO (Figure 2d) reflects the high ability to combat one of the most interrelated clinical consequences of CM (Negro & Martelletti, 2011).

In non‐converting patients, there was a significant decrease in MHDs (Figure 2a) and MSMDs (Figure 2c), which, despite the lack of reversion, leads us to believe there was a clinical benefit in both groups of patients.

The use of fremanezumab is very beneficial in the symptomatology of migraine patients, and the incorporation of new drugs with this indication, such as CGRP inhibitors, is of great importance. Major problems remain in the consultation rate, headache education and awareness in the general population, as well as the appropriate use of acute and prophylactic medication (Katsuki et al., 2023).

The main limitations of our study are its short duration, the variability in the timing of patient evaluations, and the no required use of headache daily recording, as well as its sample size, the latter because the study was limited to only one health center with patients at that same center who were being treated with fremanezumab. For future studies, multivariate analysis should be considered, as well as collaboration with other centers, with the aim of obtaining more data on fremanezumab and the reversion of CM to EM in a real population. The number of patients in whom the reversion to EM might be due to the natural course of the disease rather than treatment effectiveness cannot be estimated, which could also be assessed with more precision by conducting a longer study. Further studies are needed to assess other possible factors that act as predictors of reversion from CM to EM.

## 5 CONCLUSIONS

The results obtained in this study confirm the effectiveness of fremanezumab as a prophylactic drug in CM, providing an improvement in migraine symptomatology, with a high frequency of reversion from CM to EM, assessed for the first time in a real population and with working age patients previously treated and resistant to botulinum toxin. The improvement encompasses not only pain frequency, but also pain duration, intensity, and, secondarily, symptomatic medication consumption and its abuse, supporting the results obtained in clinical trials. Clinical improvement also applies to the quality of life of non‐converted patients. In the absence of studies with a larger sample size and longer duration, previous erenumab failure and anxiety were identified as possible detrimental predictors of reversion.

## AUTHOR CONTRIBUTIONS

**Juan Tudela‐Tomas**: Conceptualization; data curation; formal analysis; investigation; methodology; writing—original draft; writing—review and editing. **Rosa‐Maria Ramos‐Guerrero**: Conceptualization; methodology; project administration; supervision; validation; writing—review and editing. **Maria‐Eugenia Rodriguez‐Mateos**: Conceptualization; methodology; supervision; validation; writing—review and editing.

## FUNDING INFORMATION

This research received no specific grant from any funding agency in the public, commercial or not‐for‐profit sectors.

## CONFLICT OF INTEREST STATEMENT

The authors declare no conflicts of intrest.

### PEER REVIEW

The peer review history for this article is available at https://publons.com/publon/10.1002/brb3.3631

## Data Availability

The datasets used and/or analyzed during the current study are available from the corresponding author on reasonable request.
